# *ZjFAS2* is involved in the fruit coloration in *Ziziphus jujuba* Mill. by regulating anthocyanin accumulation

**DOI:** 10.3389/fpls.2023.1142757

**Published:** 2023-03-10

**Authors:** Shipeng Li, Yuanyuan Shen, Shipei Zheng, Qihang Zhu, Linfang Cai, Yian Wang, Xusheng Zhao

**Affiliations:** College of Life Science, Luoyang Normal University, Luoyang, Henan, China

**Keywords:** jujube (*Ziziphus jujuba* Mill.), fruit color, anthocyanin, WD40 repeat protein, ZjFAS2

## Abstract

Fruit color is one of the most important traits of jujube (*Ziziphus jujuba* Mill.). However, the differences in the pigments of different varieties of Jujube are not well studied. In addition, the genes responsible for fruit color and their underlying molecular mechanisms remain unclear. In this study, two jujube varieties, namely “Fengmiguan” (FMG) and “Tailihong” (TLH), were considered. The metabolites from jujube fruits were investigated using ultra-high-performance liquid chromatography/tandem mass spectrometry. Transcriptome was used to screen anthocyanin regulatory genes. The gene function was confirmed by overexpression and transient expression experiments. The gene expression was analyzed by quantitative reverse transcription polymerase chain reaction analyses and subcellular localization. Yeast-two-hybrid and bimolecular fluorescence complementation were used to screen and identify the interacting protein. These cultivars differed in color owing to their respective anthocyanin accumulation patterns. Three and seven types of anthocyanins were found in FMG and TLH, respectively, which played a key role in the process of fruit coloration. ZjFAS2 positively regulates anthocyanin accumulation. The expression profile of ZjFAS2 exhibited its different expression trends in different tissues and varieties. Subcellular localization experiments showed that ZjFAS2 was localized to the nucleus and membrane. A total of 36 interacting proteins were identified, and the possibility of ZjFAS2 interacting with ZjSHV3 to regulate jujube fruit coloration was studied. Herein, we investigated the role of anthocyanins in the different coloring patterns of the jujube fruits and provided a foundation for elucidating the molecular mechanism underlying jujube fruit coloration.

## Introduction

The combination of various pigments leads to the appearance of color in fruits. This appearance is determined by three major types of plant pigments, namely chlorophyll, carotenoids, and flavonoids (Miller et al., 2011). Anthocyanin is a class of flavonoids that are widely distributed in plant seeds as natural water-soluble pigments. These pigments are important secondary metabolites in fruits and have been identified in various fruits such as strawberries, grapes, apples, citruses, peaches, and pomegranates (Huang et al., 2018; Luo et al., 2018; Wang et al., 2018; Gao et al., 2020; Li et al., 2020; Narjesi et al., 2023).

Anthocyanin biosynthesis has been well characterized in plants (Zhang et al., 2014; Tohge et al., 2017). The anthocyanin synthesis pathway is generally divided into five stages. The first stage is the phenylpropanoid pathway, where under the action of acid lyase (PAL), cinnamic acid 4-hydroxylase (C4H), and 4-coumaroyl CoA ligase (4CL), phenylalanine undergoes a series of reactions to form 4-coumaroyl CoA. Furthermore, dihydroflavonol is synthesized from 4-coumaroyl CoA by a series of enzymes such as chalcone synthase (CHS), chalcone isomerase (CHI), and flavanone 3-hydroxylase (F3H)/flavanone 3′-hydroxylase (F3′H)/flavanone 3′-5′-hydroxylase (F3′5′H). Subsequently, anthocyanidins are converted from dihydroflavonol by dihydroflavonol 4-reductase (DFR) and anthocyanidin synthase (ANS). These anthocyanidins are ultimately modified by UDP-glucose flavonoid 3-glucosyltransferase (UFGT), rhamnosyltransferase (RT), and 3′-*O*-methyltransferase (OMT). Lastly, these anthocyanins are transported from the cytosol into the vacuoles. The anthocyanin biosynthesis is mainly regulated by three types of transcription factors, namely R2R3-MYB, bHLH, and WD40 repeat protein (Xu et al., 2015). Members of these protein families interact to form a complex (MBW complex containing MYB, bHLH, and WDR) that regulates anthocyanin biosynthesis by binding to the promoter of anthocyanin synthase (Zhang et al., 2014; Gu et al., 2019).

Studies on anthocyanin biosynthesis regulators focus more on R2R3-MYB and bHLH than WD40 repeat protein. Two types of WD40 proteins involved in anthocyanin biosynthesis regulation have been reported. The first type constitutes *TTG1* and its homologous genes, such as *AN11*, *PAC1*, *PFWD*, and *WDR1/2* (de Vetten et al., 1997; Carey et al., 2004; Hichri et al., 2010; Matus et al., 2010; Yamazaki and Saito, 2011). In Arabidopsis, TTG1 protein interacts with R2R3-MYB (PAP1/2, GL1, MYB5, MYB75, or MYB113/114) and bHLH (GL3, EGL3, or TT8) to form MBW complex, which activates the anthocyanin biosynthesis (Borevitz et al., 2001; Gonzalez et al., 2008; Gonzalez et al., 2009; Qi et al., 2011; Zhou et al., 2012). The second type constitutes *COP1* and its homologous genes. *COP1* is a key regulator that regulates light-induced anthocyanin accumulation (Li et al., 2012; An et al., 2017; Henry-Kirk et al., 2018).

Chinese jujube (*Ziziphus jujuba* Mill.) belongs to the family Rhamnaceae and is an economically and ecologically important fruit tree in Asia (Qu and Wang, 1993). Archaeological evidence suggests that the Chinese jujube has been cultivated for more than 3,000 years and has originated in China (Qu and Wang, 1993; Liu and Wang, 2009; Li et al., 2013). Chen et al reported that jujube fruit has high nutritional value and can be used as medicine and food (Chen et al., 2017). A variety of biologically active ingredients have been identified in jujube fruits, such as polysaccharides, flavonoids, phenolic acids, terpenoids, alkaloids, and vitamin C (Pawlowska et al., 2009; Gao et al., 2013; Liu et al., 2014; Chen et al., 2017; Shi et al., 2018; Zhang et al., 2020; Wen et al., 2022; Xue et al., 2022).

Jujube is an ideal natural pigment resource. Studies have shown that these pigments are flavonoids and are mainly found in the peel (Zhang et al., 2010; Choi et al., 2011; Gao et al., 2013; Kou et al., 2015). So far, 40 flavanols, 37 flavonols, 15 anthocyanins, 12 proanthocyanidins, nine dihydroflavones, eight flavanols, seven flavonoid carbonosides, five dihydroflavonols, three isoflavones, and two chalcones have been identified in different jujube fruits (Cheng et al., 2000; Bai et al., 2010; Choi et al., 2011; Choi et al., 2012; Gao et al., 2012; Collado-González et al., 2013; Du et al., 2013; Zozio et al., 2014; Chen et al., 2015; Wojdyło et al., 2016; Shi et al., 2018; Feng et al., 2020; Shi et al., 2020; Zhang et al., 2020; Xue et al., 2022). The jujube fruits studied in several studies were collected from a single variety. Therefore, there is a lack of systematic research on the jujube pigments among different varieties.

*ZjANS* and *ZjUFGT* play a crucial role in the accumulation of anthocyanin during the fruit ripening process, which is activated by *ZjMYB5*, *ZjTT8*, and *ZjTTG1* (Shi et al., 2020; Zhang et al., 2020). *ZjDFR* is involved in the regulation of postharvest fruit coloration (Kou et al., 2019). The functions of these genes have already been identified in other species. In this study, we identified a novel anthocyanin regulatory gene, *ZjFAS2*, by analyzing the physiological and biochemical characteristics and performing metabolome and transcriptome profiling, transient expression, and genetic transformation experiments. Moreover, we investigated the expression profile and subcellular localization of *ZjFAS2* and identified the proteins that interact with *ZjFAS2*. The results of this study shed light on the mechanism underlying jujube fruit coloration and provided theoretical support for the genetic improvement of jujube fruit quality.

## Materials and methods

### Plant materials

A total of 28 cultivars were obtained from the jujube germplasm resource of the Luoyang Normal University in Luoyang, Henan, China (**Supplementary Table 1**). Fruit samples of “Fengmiguan” (FMG) and “Tailihong” (TLH) cultivars were harvested at seven developmental stages on days 20, 35, 50, 65, 80, 90, and 100 after flowering and were named FMG_S1, FMG_S2, FMG_S3, FMG_S4, FMG_S5, FMG_S6, FMG_S7, TLH_S1, TLH_S2, TLH_S3, TLH_S4, TLH_S5, TLH_S6, and TLH_S7, respectively. The new sheet, secondary lateral branch, flower bud, and the seed of FMG and TLH were collected. Fruit samples of the remaining 26 cultivars were harvested at stage S6. Furthermore, the white and red sides of fruit samples were collected and named S6-W and S6-R, respectively. All the fruit samples were brought back to the laboratory in liquid nitrogen containers and stored at −80 °C until further analysis.

### Fruit color evaluation

The color of the peel of jujube fruits was measured using a colorimeter (SR-64, Shenzhen 3nh Technology Co., Ltd., Shenzhen, China), which provided information according to the Commission Internationale de system in terms of L* (brightness or lightness; 0 = black, 100 = white), a* (−a* = greenness, +a* = redness), b* (−b* = blueness, +b* = yellowness), and hue angle degree (h°) measurements. At least 10 fruits were sampled for FMG and TLH for all the stages.

### Total chlorophyll, carotenoid, and anthocyanin content analysis

Total chlorophyll and carotenoid contents were measured as described by Lichtenthaler and Wellburn (1983). Briefly, 100 mg of the sample was ground using 12 mL of 96% ethanol (v/v). The mixture was incubated under dark conditions for 3 h and then centrifuged at 10,000 rpm for 5 min. The absorbance was determined at 663 nm, 646 nm, and 470 nm using an ultraviolet–visible (UV–Vis) spectrophotometer. The total anthocyanin content was measured following the method described by Ai et al. (2016). Briefly, approximately 200 mg of the sample was ground with liquid nitrogen. The anthocyanins were extracted with 8 mL methanol–HCl (99:1 v/v). The absorbance was determined at 530 nm and 657 nm using a UV–Vis spectrophotometer. Cyanidin was used as a reference standard, and the results were expressed as cyanidin equivalents in mg/kg extract. Each sample was analyzed in triplicate. At least five fruits of each cultivar from all the stages were used.

### Metabolite identification and quantification

The samples named FMG_S1, FMG_S4, FMG_S5, FMG_S6, FMG_S7, TLH_S1, TLH_S4, TLH_S5, TLH_S6, and TLH_S7 were analyzed using ultrahigh-performance liquid chromatography-electrospray ionization-tandem mass spectrometry (UPLC-ESI-MS/MS). For each sample, three biological replicates were independently analyzed. The primary and secondary spectral data of the metabolites were detected by mass spectrometry. The identification and structural analyses of these metabolites were performed using the MWDB database (Metware Biotechnology Co., Ltd. Wuhan, China) and public databases, namely MassBank (http://www.massbank.jp/), KNAPSAcK (http://kanaya.naist.jp/KNApSAcK/), HMDB (http://www.hmdb.ca/), MoToDB (http://www.ab.wur.nl/moto/), and ChemBank (http://chembank.med.harvard.edu/compounds), PubChem (https://pubchemblog.ncbi.nlm.nih.gov/), NIST Chemistry Webbook (http://webbook.nist.gov/), and METLIN (http://metlin.scripps.edu/index.php). Metabolomics data was processed using Analyst (Version 1.6.3, Sciex, Framingham, MA, USA).

### RNA sequencing

The total RNA extracted from the samples named FMG_S1, FMG_S7, TLH_S1, TLH_S5, and TLH_S7 was collected for transcriptome analysis. For each sample, three biological replicates were independently analyzed. The RNA sequencing (RNA-Seq) was performed by Biomarker Technologies Co., Ltd. (Beijing, China). The transcriptome data were deposited in the NCBI (National Center for Biotechnology Information) Sequence Read Archive database (Accession number: SRR23330575-SRR23330589).

Differential expression analyses of two groups were performed using the DESeq R package (1.10.1). For identifying differentially expressed genes (DEGs), fold change (FC) ≥2 and false discovery rate (FDR) <0.01 were used as the screening criteria. FC represented the ratio of gene expression values in two groups. The resulting *P*-values were adjusted using Benjamini and Hochberg’s approach for controlling the FDR.

### Quantitative reverse transcription polymerase chain reaction analysis

Total RNA was extracted using a plant RNA extraction kit (Cat. No. 9769, TakaRa Biotechnology Inc., China). The quality of RNA was determined using an Agilent Bioanalyser 2100 (Agilent Technologies, Inc., USA), and the concentration was measured using an ND-2000 spectrophotometer (Nanodrop Technologies, Inc., USA). After the treatment of the RNA sample with a gDNA Eraser, the first-strand cDNA was synthesized using the PrimeScript^™^ RT reagent Kit (Cat. No. RR047A, TakaRa Biotechnology Inc., China). qRT-PCR was performed with gene-specific primers in a real-time PCR reaction system (25 µL) according to the manufacturer’s protocol (Cat. No. RR820A, TakaRa Biotechnology Inc., China). The amplification was monitored on a CFX96 real-time PCR detection system (BIO-RAD). The measurements were obtained using the relative quantification method (Livak and Schmittgen, 2001). Jujube *ACTIN* (*LOC107413530*) was used as an internal control. All analyses were based on performed using three biological samples and three replicates of each sample. The primers are listed in **Supplementary Table 2**.

### Vector construction and transformation

To construct the overexpression construct of *ZjFAS2*, the *ZjFAS2* cDNA from TLH was digested with XbaI and XhoI and then inserted into the pART-CAM vector. The appropriate constructs were confirmed by sequencing and introduced into the host cells *Agrobacterium tumefaciens* GV3101. The primers are listed in **Supplementary Table 3**.

For transgenic validation, the tobacco variety “SR1” was used as the receptor for transformation by *Agrobacterium*. Tobacco transformation was performed using a previously described method (Horsch et al., 1985; Zong et al., 2019). The transient expression assay in jujube fruits was performed on the basis of a previously described method with slight modifications (Hawkins et al., 2016). The Agrobacterium strain containing the 35S:*ZjFAS2* construct was infiltrated on the jujube fruits between stages S4 and S5 for phenotype investigation. Agrobacterium cultures carrying the empty vector served as the negative control. Patches of red color appeared 3–5 days after injection.

### Subcellular localization

The *ZjFAS2* cDNA was inserted downstream of the cauliflower mosaic virus (CaMV) 35S promoter through XhoI and EcoRI restriction enzyme sites in the frame with a green fluorescent protein (GFP) in the part-CAM-EGFP vector. The *ZjFAS2*-GFP fusion construct and nucleus/membrane protein marker (AtH2B-RFP/AtPIP2A-RFP) were cotransformed into tobacco leaves via agrobacterium-mediated transformation. The primers are listed in **Supplementary Table 3**.

### Yeast two-hybrid assays

The yeast two-hybrid cDNA library was constructed using the Make Your Own “Mate & Plate^™^” Library System (Cat. No. 630490, TakaRa Biotechnology Inc., USA). To construct the bait vector for *ZjFAS2*, the *ZjFAS2* cDNA from TLH was digested with EcoRI and BamHI and then inserted into the pGBKT7 vector. The co-transformation of yeast strain Y2HGold was performed after self-activation and toxicity detection to screen for ZjFAS2 interacting proteins. The pGADT7-jujube cDNA and pGBKT7-FAS2 were co-transformed into yeast strain Y2HGold. The yeast cells were cultured in the SD-Trp-Leu-His-Ade medium at 30 °C under dark conditions for three days. The positive colonies were selected and sequenced. The primers are listed in **Supplementary Table 3**.

### Bimolecular fluorescence complementation assays

The cDNA of *ZjFAS2* and *ZjSHV3* from TLH was digested with XbaI and KpnI and then inserted into the pCAMBIA1300S-YC and pCAMBIA1300S-YN vectors to produce the cYFP-protein and nYFP-protein constructs, respectively. The BiFC assays were performed using the method described by Guo et al. (2020). The primers are listed in **Supplementary Table 3**.

## Results

### Changes in jujube fruit color during ripening

**Figure 1:**
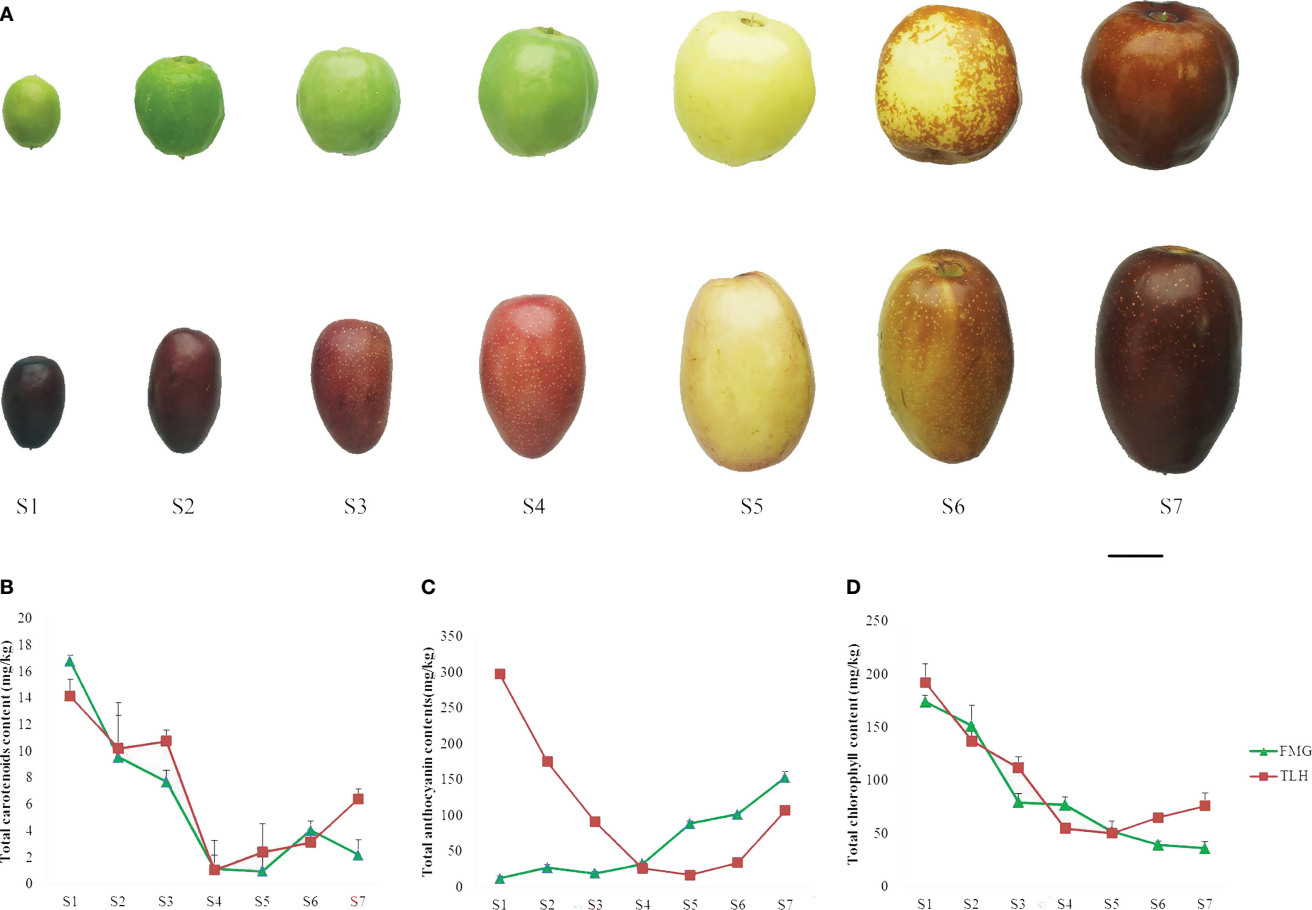
Phenotypic characterization of the jujube cultivars FMG and TLH. **(A)** Photographs of the seven stages of growth. Bars = 1 cm. **(B)** Total carotenoid content. **(C)** Total anthocyanin content. **(D)** Total chlorophyll content. Error bars indicate standard deviations (SDs) from three biological replications.

To investigate the process of the color change of the jujube fruits, we divided the fruit developmental stages into seven stages (**Figure 1A**). Harvested between 20 and 100 days after flowering, the fruit weight, length, and width of FMG ranged between 0.58–10.56 g, 13.76–29.37 mm, and 9.16–26.85 mm, respectively, and the fruit weight, length, and width of TLH ranged between 0.56–12.59 g, 16.37–38.05 mm, and 8.00–24.68 mm, respectively (**Supplementary Table 4**). Maximum fruit size and weight were recorded at stage S7. The color parameters of FMG and TLH ranged between 38.87–69.18 and 29.85–55.64 for L*, –11.10–20.68 and 5.94–19.22 for a*, 15.87–39.85 and 1.76–27.06 for b*, and 36.37–113.05 and 13.54–77.47 for h°. The h° of FMG gradually decreased with fruit development, and the maximum and minimum values were recorded at stages S1 and S7, respectively. However, the h° of TLH showed different patterns, peaking at stage S5 and then decreasing gradually (**Supplementary Table 5**). These results indicated that the fruit color of FMG and TLH differed considerably during the early stages of fruit development.

A rapid colorimetric assay was performed to evaluate the contents of anthocyanin, chlorophyll, and carotenoid. A small number of carotenoids was detected in the peels (**Figure 1B**), whereas a large number of anthocyanins and chlorophyll was detected in the peels (**Figures 1C, D**). The total anthocyanin content in FMG and TLH varied between 143.50–313.75 and 136.60–345.05 mg/kg, respectively. The content of anthocyanins in FMG gradually increased with fruit development, and the highest concentration was recorded at stage S6. However, anthocyanin content in TLH decreased to the lowest level at stage S5 and then increased gradually. The chlorophyll contents of FMG and TLH ranged between 36.25–174.22 and 50.53–192.67 mg/kg, respectively, which decreased rapidly during the ripening process. The correlation analysis showed that only the total anthocyanin content was significantly negatively correlated with h° in both FMG and TLH (**Supplementary Table 6**).

Taken together, our results indicated that the fruit color of FMG and TLH was mainly determined by anthocyanin content despite the differences in fruit coloration patterns.

### Identification of anthocyanins from the peels of jujube

**Figure 2:**
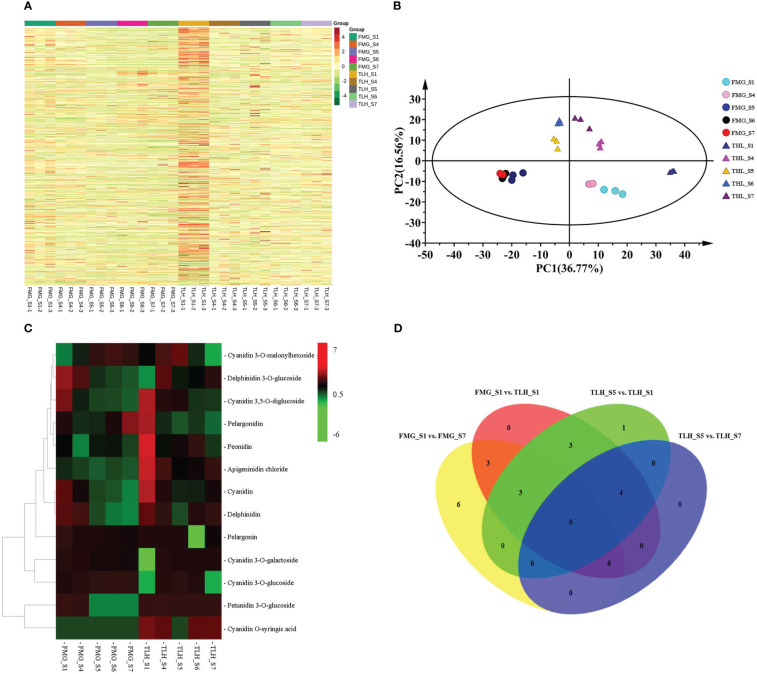
Qualitative and quantitative analysis of the metabolomics data of the peels of FMG and TLH. **(A)** Heatmap of the quantified identified metabolites. **(B)** PCA score plot of the metabolites in the FMG and TLH. **(C)** Heatmap of the quantification of the identified anthocyanins. **(D)** Venn diagram showing the number of metabolites in FMG_S1, FMG_S7, TLH_S1, TLH_S5, and TLH_S7.

To further identify the type of anthocyanins, the metabolites were investigated by UPLC-ESI-MS/MS. A total of 784 putative metabolites were identified from the peels of FMG and TLH (**Figures 2A, B**; **Supplementary Table 7**).

Of note, 12 anthocyanins were identified from the peel extracts of FMG (**Figure 2C**; **Supplementary Table 7**). The contents of three of these anthocyanins, namely cyanidin 3-*O*-glucoside, cyanidin 3-*O*-malonylhexoside, and pelargonidin, increased with the ripening process and were positively correlated with the total anthocyanin content (**Supplementary Table 8**). Conversely, nine anthocyanins, namely apigeninidin chloride, cyanidin, cyanidin 3,5-*O*-diglucoside, cyanidin 3-*O*-galactoside, delphinidin, delphinidin 3-*O*-glucoside, pelargonidin, peonidin, peonidin, and petunidin 3-*O*-glucoside, decreased or remained stable with fruit development and were negatively correlated with the total anthocyanin content (**Supplementary Table 8**).

We identified 13 anthocyanins from the peel extracts of TLH (**Figure 2C**; **Supplementary Table 7**). Nine of these anthocyanins, namely apigeninidin chloride, cyanidin, cyanidin 3,5-*O*-diglucoside, cyanidin *O*-syringic acid, delphinidin, pelargonidin, pelargonin, peonidin, and petunidin 3-*O*-glucoside, were positively correlated with the total anthocyanin content (**Supplementary Table 8**). Conversely, four anthocyanins, namely cyanidin 3-*O*-galactoside, cyanidin 3-*O*-glucoside, cyanidin 3-*O*-malonylhexoside, and delphinidin 3-*O*-glucoside, were negatively correlated with the total anthocyanin content (**Supplementary Table 8**). Multiple comparative analyses showed that apigeninidin chloride, cyanidin, cyanidin 3,5-*O*-diglucoside, cyanidin *O*-syringic acid, delphinidin, pelargonidin, and peonidin had a relatively higher content in TLH_S1, whereas apigeninidin chloride, cyanidin *O*-syringic acid, and delphinidin had a relatively higher content in TLH_S7 (**Figure 2D**). Together, these results indicated that cyanidin, cyanidin 3,5-*O*-diglucoside, pelargonidin, and peonidin play a crucial role during the early stages of the fruit coloration process, whereas apigeninidin chloride, cyanidin *O*-syringic acid, and delphinidin are involved in the complete fruit coloration process.

### Differential expression of anthocyanin biosynthesis-related genes

To evaluate anthocyanin biosynthesis at the transcriptional level, five cDNA libraries were prepared from the peels of FMG and TLH, which were subjected to RNA-Seq analysis (**Supplementary Table 9**). To verify the accuracy and reproducibility of the anthocyanin biosynthesis-related genes analysis results, 11 anthocyanin structural genes, one *R2R3-MYB* gene, five *bHLH* genes, and one *WD40* gene were randomly selected and analyzed by qRT-PCR analysis (**Supplementary Figure 1**). Pearson’s correlation coefficients further indicated that the digital transcript abundance of most genes was significantly correlated with the qRT-PCR results (**Supplementary Table 2**). These results confirmed the reliability of the RNA-Seq data.

**Figure 3:**
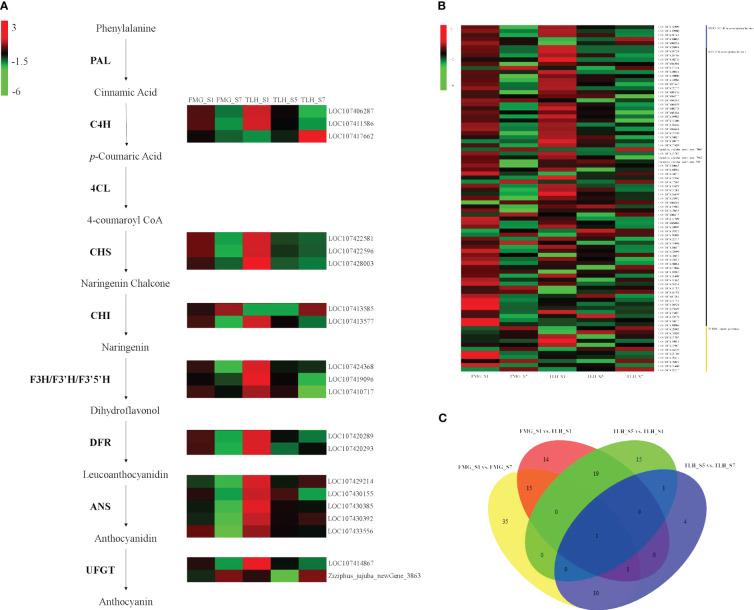
Transcriptome analyses of the peels of FMG and TLH. **(A)** Heatmap of differentially expressed anthocyanin structural genes. **(B)** Heatmap of differentially expressed *R2R3-MYB*, *bHLH*, and *WD40* genes. **(C)** Venn diagram showing the number of differentially expressed *R2R3-MYB*, *bHLH*, and *WD40* genes in FMG_S1, FMG_S7, TLH_S1, TLH_S5, and TLH_S7.

Differential expression of anthocyanin structural genes was performed. In FMG, 16 differentially expressed anthocyanin structural genes were detected in FMG_S1 and FMG_S7. Among these genes, the transcriptional levels of 14 genes decreased with fruit ripening, namely two *C4H* genes, three *CHS* genes, one *CHI* gene, one *F3H* gene, one *F3’5’H* gene, two *DFR* genes, three *ANS* genes, and one *UFGT* gene. The expression of one *CHI* gene and one *UFGT* gene was upregulated in FMG_S7 (**Figure 3A**; **Supplementary Table 10**). In TLH, 17 anthocyanin structural genes showed upregulated expression in TLH_S1, namely two *C4H* genes, three *CHS* genes, one *CHI* gene, one *F3H* gene, one *F3’H* gene, one *F3’5’H* gene, two *DFR* genes, five *ANS* genes, and one *UFGT* gene (**Figure 3A**; **Supplementary Table 11**). These 17 genes also showed upregulated expression in TLH_S1 compared with the expression in FMG_S1 (**Figure 3A**; **Supplementary Table 12**). During fruit development, one *C4H* gene, one *CHI* gene, one *F3’H* gene, and one *UFGT* gene were downregulated, and only one *UFGT* gene was upregulated in TLH_S7 (**Figure 3A**; **Supplementary Table 13**). Comparing the gene expression in FMG_S7 and TLH_S7 indicated that one *C4H* gene, one *F3H* gene, one *DFR* gene, and three *ANS* genes were upregulated, whereas only one *F3’H* gene was downregulated in TLH_S7 (**Figure 3A**; **Supplementary Table 14**).

Differential expression of *R2R3-MYB*, *bHLH*, and *WD40* genes was also detected. We detected 62 differentially expressed genes between FMG_S1 and FMG_S7. Furthermore, 47 genes were downregulated in FMG_S7, namely 11 *R2R3-MYB* genes, 33 *bHLH* genes, and three *WD40* genes; 15 genes were upregulated in FMG_S7, namely nine *bHLH* genes and six *WD40* genes (**Figure 3B**; **Supplementary Table 10**); 36 differentially expressed genes were detected between TLH_S1 and TLH_S5; and 27 genes, namely five *R2R3-MYB* genes, 20 *bHLH* genes, and two *WD40* genes, were upregulated. However, only six *bHLH* genes and three *WD40* genes were downregulated in TLH_S1 (**Figure 3B**; **Supplementary Table 11**). Conversely, only 17 differentially expressed genes were detected between TLH_S5 and TLH_S7. Four *bHLH* genes and five *WD40* genes were upregulated, whereas two *R2R3-MYB* genes and six *bHLH* genes were downregulated in TLH_S7 (**Figure 3B**; **Supplementary Table 12**). In contrast to the expression of the anthocyanin structural genes, the DEGs related to anthocyanin regulation did not show a clear trend between FMG and TLH during the same fruit developmental stages. Of note, 27 genes, namely six *R2R3-MYB* genes, 19 *bHLH* genes, and two *WD40* genes, were upregulated, and 23 genes, namely six *R2R3-MYB* genes, 14 *bHLH* genes, and three *WD40* genes, were downregulated in TLH_S1 compared with FMG_S1 (**Figure 3B**; **Supplementary Table 13**). Only six genes were differentially expressed between FMG_S7 and TLH_S7. Among these genes, one *R2R3-MYB* gene and two *bHLH* genes were upregulated, and three *bHLH* genes were downregulated in TLH_S7 (**Figure 3B**, **Supplementary Table 14**).

Multiple comparative analyses showed that only one *WD40* gene, *LOC107425703*, showed differential expression among FMG_S1, FMG_S7, TLH_S1, TLH_S5, and TLH_S7 (**Figure 3C**; **Supplementary Tables 10**-**13**). *LOC107425703* encodes a WD40 repeat protein homologous to the Arabidopsis *FASCIATA2* (*FAS2*) gene, which was hereafter designated as *ZjFAS2*. Pearson’s correlation coefficients further indicated that *ZjFAS2* expression was significantly positively correlated with the total anthocyanin content (*r* = 0.906, *P* <0.05; **Figure 3C**; **Supplementary Figure 1**). Together, these results strongly indicated that *ZjFAS2* are candidate genes affecting anthocyanin accumulation.

### *ZjFAS2* positively regulates anthocyanin accumulation

**Figure 4:**
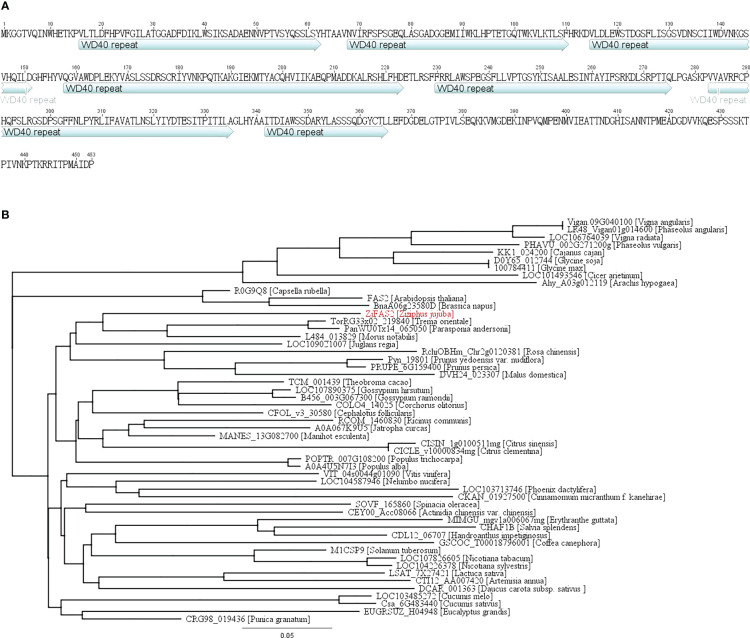
Protein sequence and phylogenetic analysis of ZjFAS2. **(A)** ZjFAS2 protein sequence. **(B)** Phylogenetic tree of the plant FAS2 protein. The lengths of the branches refer to the amino acid variation rates.

We cloned the full-length complementary DNA (cDNA) of ZjFAS2 from TLH_S1, which is 1,362 bp in length. The predicted ZjFAS2 encodes 453 amino acids and contains seven conserved WD40 repeat domains (**Figure 4A**). We collected 52 ZjFAS2 homologs from the NCBI (http://www.ncbi.nlm.nih.gov/ which encoded proteins varying from 347 to 593 amino acids in length. The FAS2 homologs can be divided into three major groups on the phylogenetic tree and the similarity of their protein sequences ranged from 63.5 to 75.9% (**Figure 4B**).

**Figure 5:**
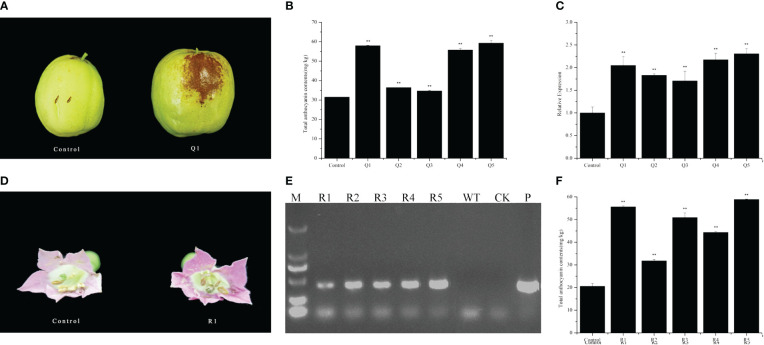
Functional analysis of *ZjFAS2*. **(A)** Fruit phenotypes of the FMG-null (Control) and ZjFAS2-transient expression fruit (Q1). **(B)** Comparison of total anthocyanin content between FMG-null (Control) and ZjFAS2-transient expression fruits (Q1-Q5). **(C)** Expression of ZjFAS2 in the FMG-null (Control) and ZjFAS2-transient expression fruits (Q1-Q5) by qRT-PCR. **(D)** Flower phenotypes of the SR1-null (Control) and ZjFAS2-transgenic lines (R1). **(E)** The examination of ZjFAS2-overexpressed lines (R1-R5), SR1 as negative control (WT), H_2_O as blank control (CK), and 35S::*ZjFAS2* pART-CAM vector (P) by PCR. **(F)** Expression of ZjFAS2 in the SR1-null (Control) and ZjFAS2-transgenic lines (R1-R5) by qRT-PCR. ** indicates significantly different at *P* < 0.01. Error bars indicate standard deviations (SDs) from three biological replications.

To validate the genetic function of *ZjFAS2*, we cloned the cDNA of ZjFAS2 into the pART-CAM vector and introduced the construct into jujube and tobacco via agrobacterium-mediated transformation. ZjFAS2 overexpression significantly increased anthocyanin accumulation in jujube fruits (**Figures 5A–C**). Furthermore, *ZjFAS2* expression was significantly positively correlated with the total anthocyanin content (*r* = 0.845, *P* <0.05). In the tobacco transgenic plants, we found that the petals of transgene-positive plants carrying ZjFAS2 had significantly higher total anthocyanin content than the petals of transgene-negative plants (**Figures 5D–F**). These results provided evidence that *ZjFAS2* plays a major role in anthocyanin accumulation.

### Expression pattern and subcellular localization of *ZjFAS2*

**Figure 6:**
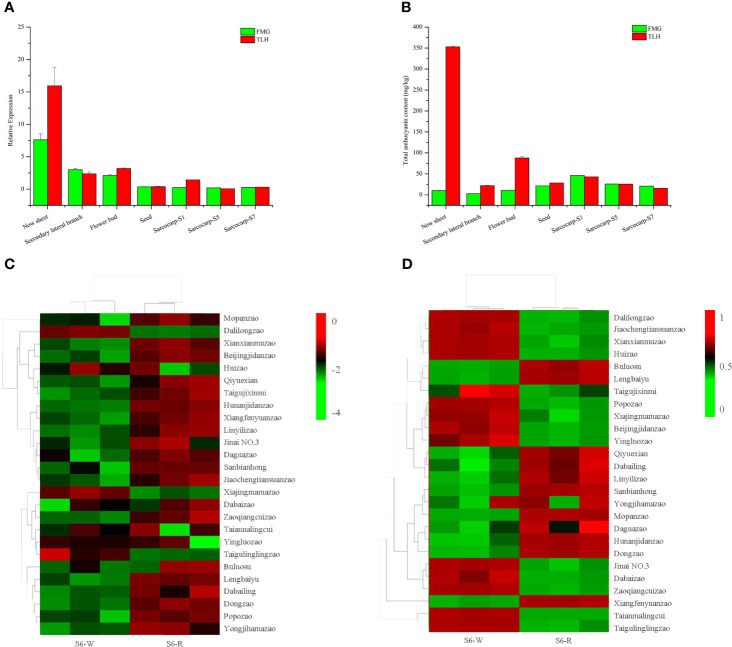
Expression patterns of *ZjFAS2*. **(A)** qRT-PCR analysis of *ZjFAS2* in Secondary lateral branch, flower bud and seed of FMG and TLH. **(B)** Total anthocyanin content n Secondary lateral branch, flower bud and seed of FMG and TLH. **(C)** Heatmap of *ZjFAS2* expression level in S6-W and S6-R from 26 varieties. **(D)** Heatmap of total anthocyanin content in S6-W and S6-R from 26 varieties. Error bars indicate standard deviations (SDs) from three biological replications.

Our previous study indicated that *ZjFAS2* expression and the total anthocyanin content in purple leaves are significantly higher than those in green leaves (Li et al., 2021). To further evaluate the expression profile of *ZjFAS2*, qRT-PCR and pigment analysis were performed in various organs of TLH and FMG (**Figures 6A, B**). The total anthocyanin content in the new sheet, secondary lateral branch, flower bud, and seed of TLH was higher than that in the respective tissues of FMG. Conversely, the total anthocyanin content in the sarcocarp of TLH was lower than that in the sarcocarp of FMG. Pearson’s correlation coefficients further indicated that *ZjFAS2* expression was significantly positively correlated with the total anthocyanin content (*r* = 0.848, *P* <0.01). To examine the expression profile of *ZjFAS2* in further detail, the fruits from the semi-red period of 26 cultivars were used in the analysis. Compared with the S6-W, the total anthocyanin content showed a higher concentration in the S6-R of 11 varieties, a lower concentration in the S6-R of 13 varieties, and a similar concentration in the S6-R of two varieties (**Figure 6C**). However, *ZjFAS2* expression showed different patterns. Compared with the *ZjFAS2* in S6-W, *ZjFAS2* showed upregulated expression in the S6-R of 18 varieties, lower expression in the S6-R of three varieties, and similar expression in the S6-R of five varieties (**Figure 6D**).

**Figure 7:**
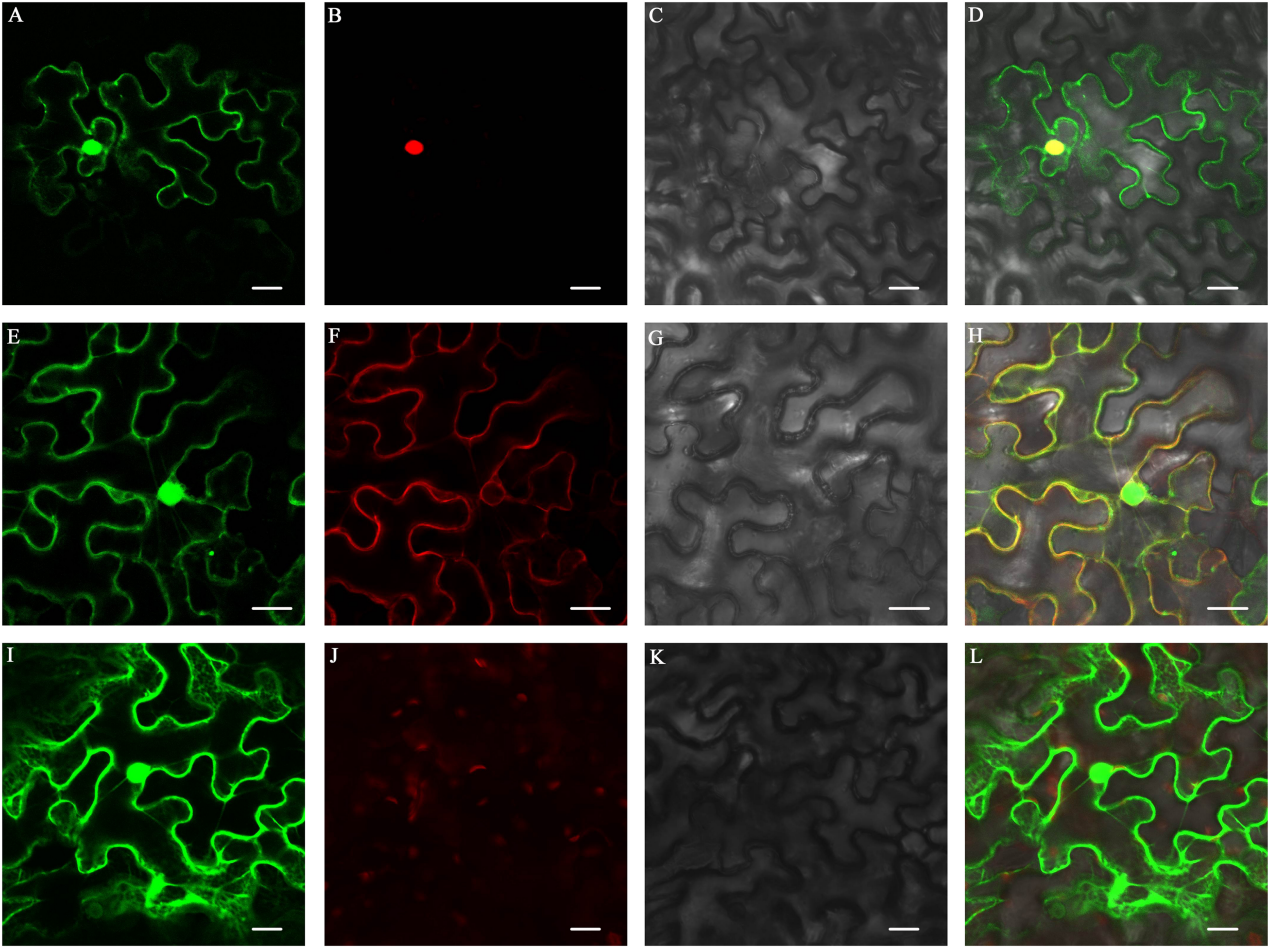
Subcellular localization of ZjFAS2. Colocalization of ZjFAS2-GFP **(A)** and AtH2B-RFP **(B)** in the tobacco leaves. The bright-field image **(C)** and merged image **(D)** are also shown. Colocalization of ZjFAS2-GFP **(E)** and AtPIP2A-RFP **(F)** in the tobacco leaves. The bright-field image **(G)** and merged image **(H)** are also shown. **(I)** The green fluorescent signal of null-GFP in the tobacco leaves. **(J)** The red fluorescent signal in the same tobacco leaves as **(I)**. **(K)** The same tobacco leaves as **(I)** under bright field. **(L)** The merged image of **(I)**, **(J)** and **(K)**. Bars = 20 μm.

To detect the subcellular localization of ZjFAS2, we constructed a GFP-ZjFAS2 fusion, whose expression was driven by the CaMV 35S promoter. The transient expression experiment in tobacco epidermal cells showed that the GFP-ZjFAS2 fusion protein was colocalized with the markers in both the nucleus and membrane (**Figure 7**).

### Identification of proteins interacting with *ZjFAS2*

**Figure 8:**
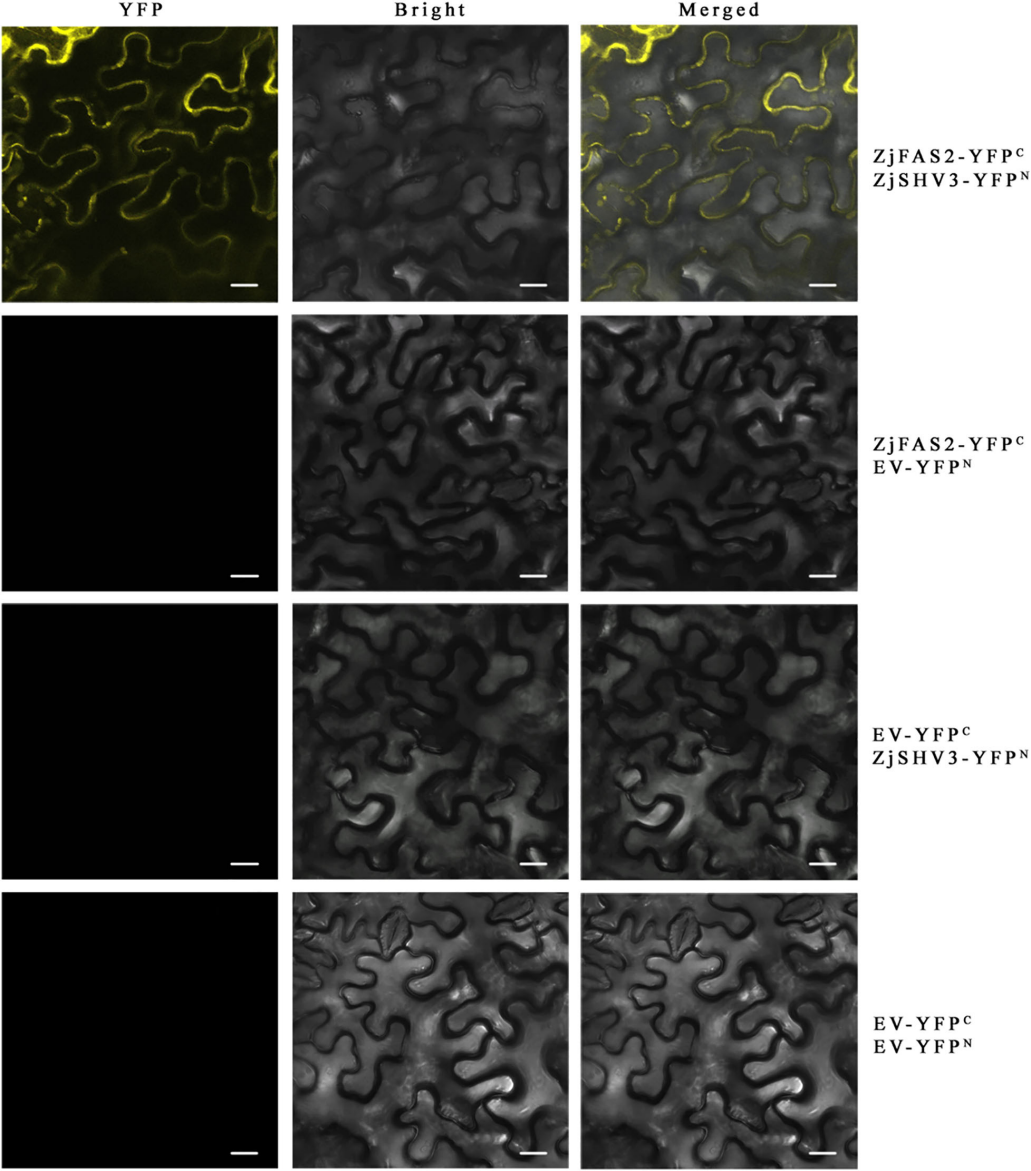
BiFC assay verifies the interaction between ZjFAS2 and ZjSHV3 in the tobacco leaves. Bars = 20 μm.

No study has reported that *FAS2* is involved in the regulation of anthocyanin accumulation. To elucidate the regulatory mechanism underlying the role of *ZjFAS2* on jujube fruit coloration, 36 interacting proteins were identified by the yeast two-hybrid library screens (**Supplementary Figure 2**; **Supplementary Table 15**). Notably, one of the ZjFAS2 interactors, the lycerophosphodiester phosphodiesterase GDPDL3-like (LOC107434323) is highly homologous with Arabidopsis SHAVEN3 (AtSHV3). The *shv3* mutant of *Arabidopsis thaliana* can increase anthocyanin accumulation (Hayashi et al., 2008). Moreover, we determined the interaction of ZjFAS2 and ZjSHV3 *in vivo* by the BiFC assay (**Figure 8**). Therefore, we believe that ZjFAS2 may interact with ZjSHV3 to regulate jujube fruit coloration.

## Discussion

The fruit color of FMG was mainly determined by chlorophyll during the early stages of fruit development and was mainly determined by anthocyanins during the late stages of fruit development on the basis of the color phenotype and pigment (**Figures 1C, D**). Compared with FMG, “Junzao” exhibited a similar fruit color change process and chlorophyll change patterns but different anthocyanin change patterns (Shi et al., 2018; Shi et al., 2019; **Figures 1B–D**). Lutein is considered to determine the fruit color of “Junzao” during the full maturity stage (Shi et al., 2018). Our results indicated that the anthocyanin content differs significantly among the varieties during the semi-red period, which is consistent with the results of a previous study (Zhang et al., 2020; **Figure 6C**). Moreover, in contrast to the fruit color of FMG and “Junzao,” the fruit color of TLH is determined by anthocyanin content during both the early and late stages of fruit development and was determined by both chlorophyll and anthocyanin contents during the middle of fruit development (Shi et al., 2018; Shi et al., 2019; **Figures 1C, D**). Our results showed that the fruit color of different varieties is determined by different pigments in jujube, which is consistent with the results of previous studies.

Multiple anthocyanin structural genes were upregulated in TLH_S1, which resulted in significantly higher total anthocyanin content in TLH than in FMG (**Supplementary Table 12**). Furthermore, FMG and TLH exhibit different fruit coloring patterns. However, most of the transcriptional levels of anthocyanin biosynthetic genes showed the same expression trends with ripening (**Figure 3A**). In both FMG and TLH, most of the anthocyanin structural genes, namely *C4H*, *CHS*, *CHI*, *F3H*, *F3’H*, *F3’5’H*, *DFR*, *ANS*, and *UFGT*, showed higher transcriptional levels during the immature stage but were downregulated during the ripening period, and only one *CHI* gene and one *UFGT* gene were upregulated during the ripe stage, which is consistent with a previous study (Shi et al., 2020). A previous study also showed similar results wherein the anthocyanin structural genes were highly active during the white period (stage S5) but were gradually silenced over the ripening period (stage S7), and only three *UFGT* genes were gradually activated during the ripening period in “Dongzao” (Zhang et al., 2020). Therefore, the expression pattern of anthocyanin structural genes was not limited to the late fruit development period but was rather involved in the complete fruit development period among the different varieties. This indicates that the increased expression of early biosynthetic genes could promote anthocyanidin accumulation in early fruit development. Furthermore, these anthocyanidins are gradually modified to anthocyanins by *UFGT* during the fruit ripening process.

In Arabidopsis, *FAS2* maintains the morphologies of stem, leaf, and flower and the organization of shoot and root apical meristems (Reinholz, 1966; Ottoline Leyser and Furner, 1992; Kaya et al., 2001). Furthermore, FASCIATA 1 (FAS1), FAS2, and MULTICOPY SUPPRESOR OF IRA1 (MSI1) form the chromatin assembly factor-1 (CAF-1) complex, which is required for DNA replication and nucleotide excision repair (Endo et al., 2006; Exner et al., 2006; Schönrock et al., 2006; Mozgova et al., 2010; Picart-Picolo et al., 2020). Through yeast two-hybrid screening, the interaction of FAS1 and FAS2 was verified in jujube (**Supplementary Table 15**). Notably, to the best of our knowledge, the role of *FAS2* in the regulation of anthocyanin accumulation was reported for the first time. Surprisingly, unlike the reported WD40 proteins, we did not find that FAS2 forms a complex with MYB and bHLH in the nucleus to regulate anthocyanin accumulation. However, the results of subcellular localization and BiFC assays supported that ZjFAS2 may interact with ZjSHV3 to regulate jujube fruit coloration on the cell membrane (**Figures 7**, **8**). Therefore, our results revealed a novel gene function of *FAS2* and a new model of anthocyanin regulation.

Fruit color is modulated by environmental and biological factors (An et al., 2018; Henry-Kirk et al., 2018; Behzadi et al., 2021; Mahmoudian et al., 2021). Carbohydrate is an important factor affecting anthocyanin accumulation and sucrose has the most significant regulatory effect (Gu et al., 2019). Among different jujube varieties, sucrose, glucose, and fructose contents show different trends, but the total carbohydrate content gradually increases during fruit maturation (Guo et al., 2015; Song et al., 2019; Feng et al., 2020). In this study, 23 carbohydrates were identified from the peel extracts of FMG and TLH (**Supplementary Table 7**). Among them, five carbohydrates, namely Ribulose-5-phosphate, D(+)-Melezitose O-rhamnoside, Maltotetraose, Trehalose 6-phosphate, and D(+)-Melezitose, were positively correlated with the total anthocyanin content in FMG. Only one carbohydrate, D(−)-Threose, was positively correlated with the total anthocyanin content in TLH. Furthermore, sucrose exhibited high concentrations during the color change period in both FMG and TLH. The carbohydrates may play a crucial role in the jujube fruit coloration process.

**Figure 9:**
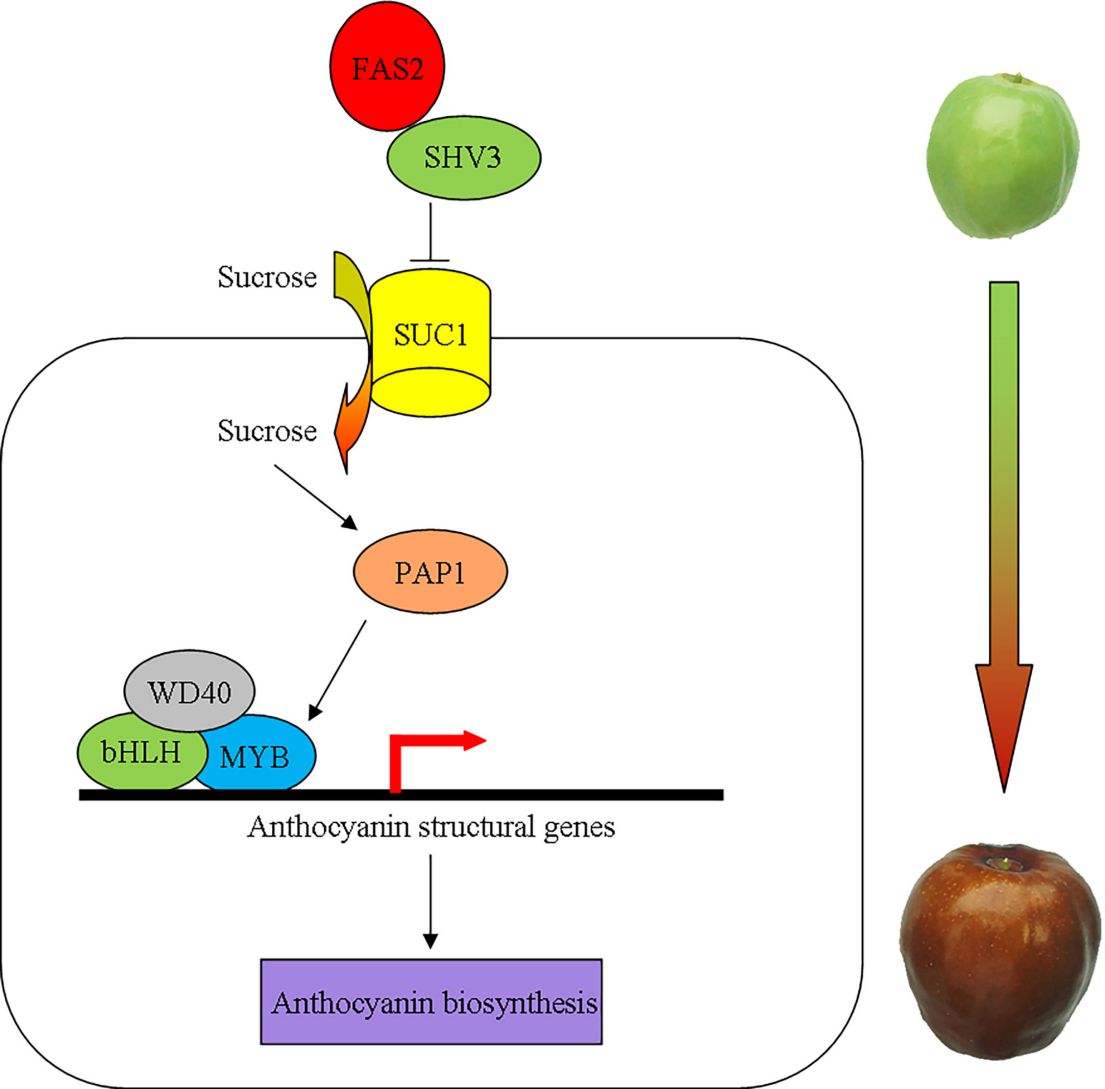
A schematic model for the possible molecular mechanism of anthocyanin accumulation regulated by *FAS2*. *FAS2* enhances the sucrose accumulation *via* the plasma membrane sucrose-proton symporter SUC1 by inhibiting SHV3 protein activity. The higher sucrose concentration causes an increase in the expression of *MYB75* genes. MYB75 interacts with bHLH and WD40 to form the MBW complex positively regulating the expression of anthocyanin structural genes.

The *SHV3* is involved in cellulose biosynthesis, hypocotyl elongation, and anthocyanin accumulation (Hayashi et al., 2008; Yeats and Somerville, 2016a; Yeats et al., 2016b). The deficiency of *SHV3* and its paralogs (*SVL1*)increases sucrose accumulation *via* the plasma membrane sucrose-proton symporter SUC1 (Yeats et al., 2016b). Conversely, sucrose acts as a signaling molecule that promotes SUC1 phosphorylation and protein activity (Niittyla et al., 2007; Yeats and Somerville, 2016a; Yeats et al., 2016b). Furthermore, *SUC1* and an R2R3-MYB transcription factor, *MYB75*/*PAP1*, are required for sucrose-induced anthocyanin accumulation (Holton and Cornish, 1995; Teng et al., 2005; Sivitz et al., 2008; Lasin, 2019). In plants with *suc1* mutant, anthocyanin structural genes that are downstream of *MYB75* were downregulated, which resulted in decreased anthocyanin accumulation (Sivitz et al., 2008). To summarize, we propose a working model of the molecular mechanism of anthocyanin accumulation regulated by *FAS2* (**Figure 9**). When the carbohydrate content reaches a certain threshold, the FAS2 increases the sucrose accumulation *via* the plasma membrane sucrose-proton symporter SUC1 by inhibiting SHV3 protein activity, resulting in sucrose-induced anthocyanin accumulation.

## Data availability statement

The datasets presented in this study can be found in online repositories. The names of the repository/repositories and accession number(s) can be found in the article/**Supplementary Material**.

## Author contributions

SL designed and supervised the study, and conducted most of the experiments. YS performed fruit color measurement and pigment content analysis. SZ participated in anthocyanin identification and quantification. QZ participated in genetic transformation. LC and YW participated in expression pattern. SL and SZ initiated the project. SL analyzed the data and wrote the manuscript. All authors contributed to the article and approved the submitted version.
