# Supplementary figures and images for: ZjFAS2 is involved in the fruit coloration in Ziziphus jujuba Mill. by regulating anthocyanin accumulation
Source: Front Plant Sci. 2023 Mar 10;14:1142757. doi: 10.3389/fpls.2023.1142757 (PMC10036858; doi:10.3389/fpls.2023.1142757)

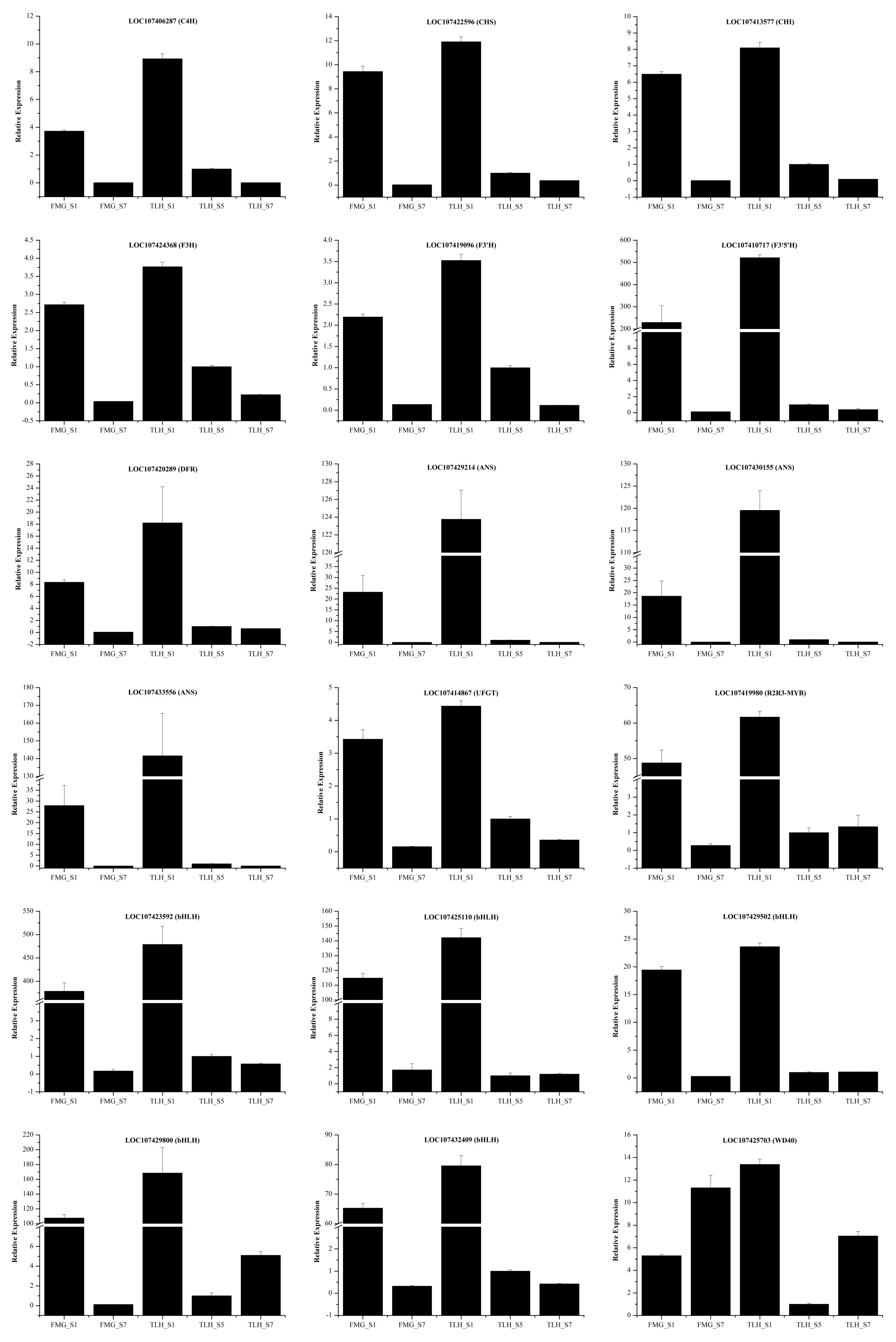

Supplement: Supplementary file 1 [file Image_1.tif]

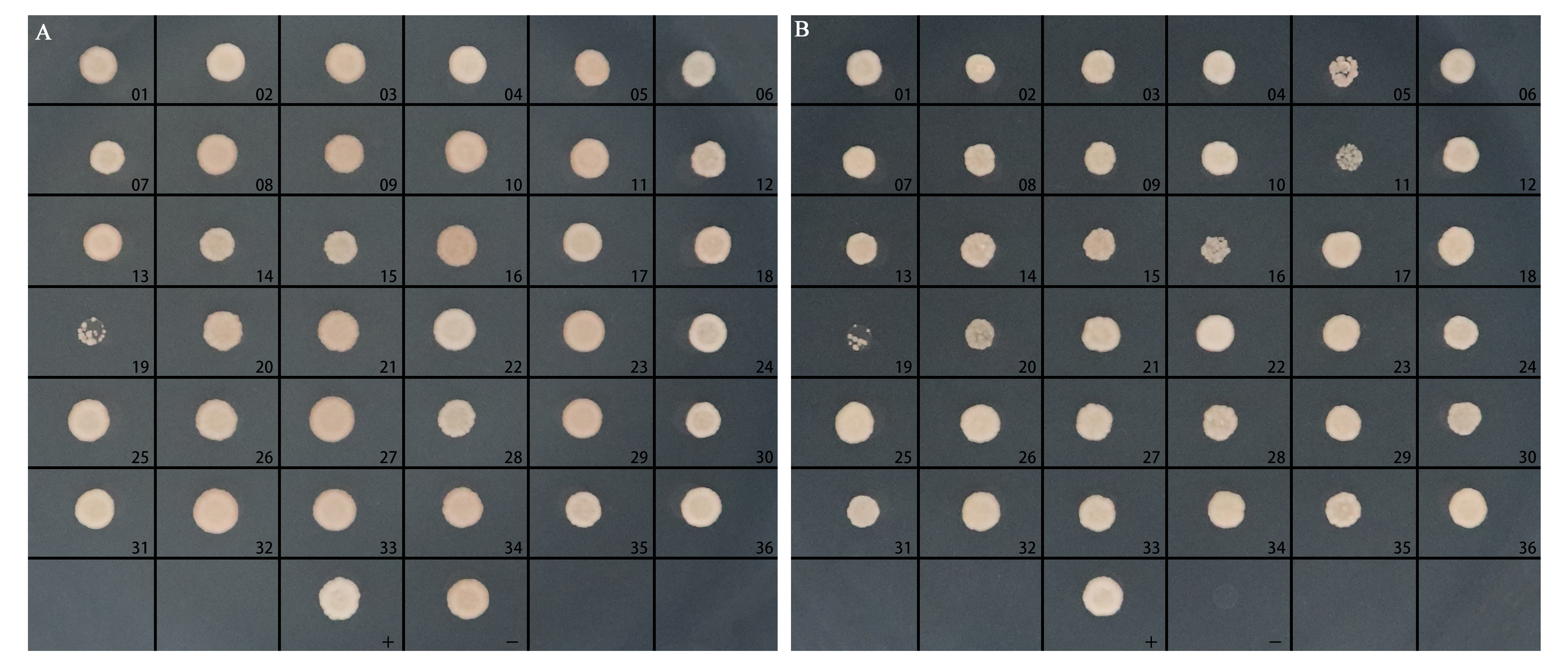

Supplement: Supplementary file 2 [file Image_2.tif]
